# An Exploration of the Relations Between Self-Reported Gender Identity and Sexual Orientation in an Online Sample of Cisgender Individuals

**DOI:** 10.1007/s10508-018-1239-y

**Published:** 2018-07-03

**Authors:** Roi Jacobson, Daphna Joel

**Affiliations:** 1 School of Psychological Sciences, Tel-Aviv University, POB 39040, Tel-Aviv, Israel; 2 Sagol School of Neuroscience, Tel-Aviv University, POB 39040, Tel-Aviv, Israel

**Keywords:** Gender identity, Sexual orientation, Bisexuality

## Abstract

The present study explored the relations between self-reported aspects of gender identity and sexual orientation in an online sample of 4756 cisgender English-speaking participants (1129 men) using the Multi-Gender Identity Questionnaire and a sexual orientation questionnaire. Participants also labeled their sexual orientation. We found a wide range of gender experiences in the sample, with 38% of the participants feeling also as the “other” gender, 39% wishing they were the “other” gender, and 35% wishing they had the body of the “other” sex. Variability in these measures was very weakly related to sexual orientation, and these relations were gender-specific, being mostly U shaped (or inverted-U shaped) in men and mostly linear asymptotic in women. Thus, in women, feeling-as-a-woman was highest in the exclusively heterosexual group, somewhat lower in the mostly heterosexual group, and lowest in the bisexual, mostly homosexual, and exclusively homosexual groups, which did not differ, and the reverse was true for feeling-as-a-man (i.e., lowest in the exclusively heterosexual group and highest in the bisexual, mostly homosexual, and exclusively homosexual groups). In men, feeling-as-a-man was highest at both ends of the sexual orientation continuum and lowest at its center, and the reverse was true for feeling-as-a-woman. Similar relations were evident also for the other aspects of gender identity. This study adds to a growing body of literature that questions dichotomous conventions within the science of gender and sexuality. Moreover, our results undermine the tight link assumed to exist between sexual and gender identities, and instead posit them as weakly correlated constructs.

**Electronic supplementary material:**

The online version of this article (10.1007/s10508-018-1239-y) contains supplementary material, which is available to authorized users.

## Introduction

Common scientific scholarship typically postulates direct relations between biological sex,^1^ gender identity, and sexual orientation (Rees-Turyn, Doyle, Holland, & Root, 2008). These different constructs are assumed to be interrelated and congruent so that each of two biological sexes (male/female) serves as a substrate for the development of one of two distinct gender identities (boy/man, girl/woman) (Diamond & Butterworth, 2008) and a sexual attraction toward the opposite sex. Moreover, it is frequently believed that atypicality in one rung (sex, gender identity, or sexuality) predicts atypicality in the other two (Jordan-Young & Rumiati, 2012; Ponse, 1978; Richardson, 2007).

Two main areas of investigation fuel these beliefs. The first is findings of higher rates of gender dysphoria and non-heterosexual tendencies in some types of physical intersex conditions compared to the general population (Cohen-Kettenis, 2005; Hines, Brook, & Conway, 2004; Jürgensen et al., 2013; Meyer-Bahlburg, 2005; Praveen et al., 2008; Zucker, 1999). The second line of evidence is findings of higher levels of non-heterosexual tendencies in adults who were gender nonconforming in childhood, higher levels of childhood gender nonconformity and gender dysphoria in non-heterosexual adults, and higher levels of adult gender nonconformity in non-heterosexual adults (e.g., Bailey, 2003; Bailey, Dunne, & Martin, 2000; Bailey et al., 2016; Bailey & Zucker, 1995; Deogracias et al., 2007; Drummond, Bradley, Peterson-Badali, & Zucker, 2008; Green, Roberts, Williams, Goodman, & Mixon, 1987; Lippa, 2005a, 2005b, 2008; Peters, Manning, & Reimers, 2007; Rahman, Abrahams, & Wilson, 2003; Rieger, Linsenmeier, Gygax, & Bailey, 2008; Rieger, Linsenmeier, Gygax, Garcia, & Bailey, 2010; Rule, Ambady, Adams, & Macrae, 2008; Singh et al., 2010, 2011; Steensma, van de Ende, Verhulst & Cohen-Kettenis, 2013; Wallien & Cohen-Kettenis, 2008; Zuger, 1988).

Other studies, however, challenge the binary view of gender identity and sexuality that underlies the prevalent understanding of the relations between sex, gender identity, and sexuality. Thus, studies on gender identity have shown that many transgender and cisgender individuals perceive their gender identity in ways that transcend the normative either/or binary notion of gender (Bockting, 2008; Bornstein, 1995; Corbett, 2009; Cromwell, 2006; Diamond & Butterworth, 2008; Joel, Tarrasch, Berman, Mukamel, & Ziv, 2013; Martin, Andrews, England, Zosuls, & Ruble, 2017; Sanger, 2008). For example, a study of gender identity in a large Israeli sample of cisgender individuals found that around 35% of the participants felt to some extent also as the “other” gender (Joel et al., 2013). A recent study in 6–11-year-old children in the U.S. similarly found that around 30% felt highly similar to both girls and boys (Martin et al., 2017). A national survey in the U.S. has recently found that less than one-third of women and men had rated themselves as very feminine or very masculine, respectively, 7% rated themselves identically on the feminine and masculine items, and 4% reported a lower score on the sex-”typical” scale than on the sex-”atypical” scale (Magliozzi, Saperstein, & Westbrook, 2016).

Challenges to the binary view of sexuality arise from findings in cisgender individuals that sexual orientation is a multi-dimensional construct (Vrangalova & Savin-Williams, 2012) that does not fit distinct categories (reviewed in Savin-Williams, 2016), and that may change across the life span (Diamond, Bonner, & Dickenson, 2015; Dickson, Paul, & Herbison, 2003; Meier, Pardo, Labuski, & Babcock, 2013; Mock & Eibach, 2012; Ott, Corliss, Wypij, Rosario, & Austin, 2011).

Research on sexuality in individuals with non-typical gender identities does not support a simple relation between gender identity and sexuality. Thus, transgender individuals, like cisgender individuals, may identify with all possible categories on the spectrum of sexual orientation (Auer, Fuss, Höhne, Stalla, & Sievers, 2014; Kuper, Nussbaum, & Mustanski, 2012; Meier et al., 2013; Lawrence, 2010; Rowniak & Chesla, 2013), and some gender dysphoric individuals change their sexual orientation during transition (Bockting, Benner, & Coleman, 2009; De Cuypere et al., 2005; Dozier, 2005; Lawrence, 2005; Meier et al., 2013; Schleifer, 2006). One study, for example, reported that among FtM (Female-to-Male) individuals, 52% were attracted to both men and women, and half of those previously attracted exclusively to women have experienced a shift in attraction (Meier et al., 2013). Heterosexual and homosexual MtF (Male-to-Female) individuals have been shown to differ on average on several variables, such as additional aspects of sexuality, treatment outcome, and comorbid psychopathology (reviewed in Lawrence, 2010), but when gender identity was assessed in males with gender dysphoria, there were no significant differences between groups with different sexual orientations (Deogracias et al., 2007).

A study on the relations between sexuality and gender identity in cisgender individuals found in a large Israeli sample that sexual orientation was not a major contributor to the perception of gender identity (Joel et al., 2013). Moreover, Joel et al. introduced another level of complexity to the discussion of the relations between gender identity and sexual orientation, by demonstrating that different aspects of gender identity differ in terms of their relations with sexual orientation.

Gender identity was assessed in Joel et al.’s (2013) study using the Multi-Gender Identity Questionnaire (Multi-GIQ), developed on the basis of existing questionnaires for the assessment of gender dysphoria in clinical populations. The Multi-GIQ includes items that assess feeling as a woman, feeling as a man, feeling as both a man and a woman, feeling as neither, contentment with affirmed gender, the wish to be the “other” gender, contentment with one’s sexed body, the wish to have the body of the “other” sex, and compliance with gender norms in clothing. In contrast to previous questionnaires, such as the Gender Identity/Gender Dysphoria Questionnaire for Adolescents and Adults (Deogracias et al., 2007), the Multi-GIQ assesses different aspects of gender identity as a woman and as a man, without a priori assuming that some of these aspects (e.g., wishing-to-be-the-“other”-gender, feeling-as-the-“other”-gender) are dysphoric, or labeling them as nonconforming.

The purpose of the present study was to use the Multi-GIQ to study gender identity in an English-speaking sample of heterosexual and non-heterosexual cisgender individuals, as well as to explore further the relations between gender identity and sexual orientation by using a finer assessment of participants’ sexual orientation. Specifically, whereas Joel et al. (2013) compared heterosexual to homosexual/bisexual individuals, the present study used five sexual orientation categories (exclusively heterosexual, mostly heterosexual, bisexual, mostly homosexual, and exclusively homosexual). Additionally, the sexuality questionnaire used in the present study included two questions that assessed the level of sexual attraction to men and to women, in addition to questions assessing the frequency of same- and “other”-sex sexual fantasies, romantic relationships, and sexual relations. We would like to note that although relying on questionnaires that use a binary conception of gender may limit the representation of identities that transcend the binary conception, it enabled us to examine to what extent people who self-identify using the “normative” gender identities, man/woman, may deviate from the assumed dichotomous and coherent perceptions.

## Method

### Participants

Participants were recruited to complete an Internet questionnaire with special effort to recruit participants from sexual minority groups (“minority” in terms of the proportion in the population). No means were taken to guarantee random sampling of the population. Invitations were sent to several groups and organizations that concentrate on LGBT issues and posted on relevant online forums. Invitations were also posted on the Facebook profiles of the researchers. The invitation included an explicit request to forward the invitation to as many people as possible. In the present study, we included only participants from English-speaking countries, who identified with their birth-assigned gender in both childhood and adulthood (women: participants who chose the following answers for these three demographic questions: Sex at birth: female, Reared as: girl, Current gender: woman, and men: participants who chose male, boy, and man in response to these questions).

Table 1 shows the distribution of participants according to self-affirmed gender and self-affirmed sexual orientation. As the number of participants in the “asexual” and “other” categories was small, we did not include participants from these categories in the study. In addition, we grouped all the participants in the “pansexual” and “bisexual” categories into one category titled bisexual, so that the variable sexual orientation included five categories: exclusively heterosexual, mostly heterosexual, bisexual, mostly homosexual, and exclusively homosexual. Tables 2 and 3 show statistics of the different demographic variables (see Tables 1s and 2s in Supplementary materials for a detailed gender × sexual orientation analysis of the variables). There were several differences, mostly small, between men and women in age, education level, religiosity level, childhood living area, current living area, and feminist attitudes (“Do you hold feminist views?”). The item about queer attitudes (“Do you hold queer views?”) was not included in the final analysis because it yielded extreme standardized residuals (ranging from − 10.3 to 12.6), suggesting outliers.

**Table 1:** Distribution of participants according to gender and sexual orientation

	Exclusively heterosexual	Mostly heterosexual	Bisexual	Mostly homosexual	Exclusively homosexual	Pansexual	Asexual	Other
*Gender*								
Men	521 (44.7%)	203 (17.4%)	58 (5.0%)	78 (6.7%)	239 (20.5%)	30 (2.6%)	14 (1.2%)	22 (1.9%)
Women	1229 (32.8%)	1023 (27.3%)	544 (14.5%)	219 (5.8%)	332 (8.9%)	280 (7.5%)	57 (1.5%)	64 (1.7%)

**Table 2:** Results of chi-square analysis of current living area, childhood living area, and feminist views for men and women

	Men	Women	*χ* ^2^
Current living area (*N* = 4708)			
Urban	46.7% (521)	45.1% (1621)	.91
Suburban	39.9% (445)	41.3% (1485)	
Rural	13.4% (150)	13.5% (486)	
Childhood living area (*N* = 4731)			
Urban	24.1% (271)	22.4% (808)	1.41
Suburban	52.0% (585)	52.9% (1908)	
Rural	23.9% (269)	24.7% (890)	
Feminist views (*N* = 4736)			
Yes	48.5% (545)↓	76.2% (2745)↑	341.82*
To some extent	39.6% (445)↑	20.6% (745)↓	
No	11.8% (133)↑	3.2% (114)↓	

↑observed frequency is higher than expected frequency, ↓observed frequency is lower than expected frequency, *p < .01

**Table 3:** Results of ANOVA or Mann–Whitney *U* test of age, religiosity, and education levels

	Men	Women	Result of statistical analysis
Age			*F*(1, 4733) = 18.27, *p* < .001, *d* = 0.14
M	35.04	33.05	
SD	14.68	13.43	
Range	16–89	16–82	
Religiosity level			*U* = 1968482.0, *p* = .32
Median	1	1	
IR	1	1	
Education level			*U* = 1988991.5, *p* = .20
Median	6	6	
IR	2	2	

*IR* interquartile range, *SD* standard deviation

*p < .01

Of the 6194 cisgender men and women who responded to the original questionnaire, we included only 4921 who came from countries in which English was the native language (Table 3). In addition, we excluded eight participants who identified as cisgender but whose responses in the questionnaires suggested otherwise (e.g., a cisgender identified woman who had not thought of herself as a woman in the past 12 months but did think of herself as a man). With the reduction of 157 “asexual” and “other” identified participants, 4756 participants were included in the final analysis.

### Procedure

The questionnaire was administered over the Internet. On the first page, participants were informed about the research aims (studying how people perceive their gender identity) and were assured as to the anonymity of their contribution. Ways of contacting the researchers were presented. By pressing “continue,” the Multi-GIQ questionnaire was displayed and was followed by the sexuality and demographic questionnaires. All questions from the GIQ and sexuality questionnaires were presented one at a time and the questions from the demographic questionnaire were presented simultaneously. Participants could press “next” without answering a question but couldn’t go back and change their chosen answers.

### Measures

The Multi-GIQ includes 24 questions that are either gender-neutral or presented twice, once as if meant for a male participant and once as if meant for a female participant (Joel et al., 2013, see Appendix for the full text of the questionnaire). Answers were marked over a 5-point Likert scale ranging from “Never” (0) to “Always” (4). A “Not relevant” item was added where necessary (e.g., the question: “In the past 12 months, have you had the wish or desire to be a man?” is not relevant for men). On the basis of participants’ responses, 13 variables were analyzed, of which three were created on the basis of theoretical considerations by averaging the scores of two questions (“Not relevant” was treated as a missing value) and the rest were scores on single items of the Multi-GIQ. The 13 variables were: “feeling-as-a-woman” (Q3 and Q14, *r* = .88); “feeling-as-a-man” (Q4 and Q13, *r* = .91); “feeling-as-both-genders” (Q15 and Q16, *r* = .75); “feeling-as-neither-gender” (Q17); “satisfied-being-a-woman/man” (Q1 and Q2, respectively); “wish-to-be-a-man/woman” (Q20 and Q21, respectively); “dislike-my-body-due-to-its-female/male-form” (Q22 and Q23, respectively), “wish-to-have-the-body-of-the-other-sex” (Q24). Items Q11 and Q12 were originally combined in Joel et al.’s (2013) study to assess “gender performance” (together with another item that assessed gender nonconforming use of the Hebrew language), but their correlation (*r* = .54) in the present study did not support combining them into a single variable. Because these were the only items in the present study that assessed gender nonconforming behaviors, we included them in the present study but analyzed them separately. Six items, which were used in Joel et al.’s study to measure “gender as performance” (perceiving one’s assigned gender as performative), were not included in the final analysis because of low reliability (*α* = .58 and *α* = .63 for men and women, respectively). Similarly, Items Q18 and Q19, which were included in Joel et al. in the satisfied-being-a-man/woman variables, were not used in the present analysis because of low correlations with the items that directly tested these (“…,have you felt satisfied being a woman/man”, *r* = .12 and *r* = .25 in men and women, respectively).

The Sexual Orientation Questionnaire includes 8 questions: In the past 12 months, have your romantic relationships been with men? (Always, Often, Sometimes, Rarely, Never, I was not involved in romantic relationships in the past 12 months); in the past 12 months, how often did you have erotic fantasies with a man (or men) as the object (or objects) of fantasy? (Very often, Often, Sometimes, Rarely, Very rarely, Never); in the past 12 months, when you had sex, was it with men? (Always, Often, Sometimes, Rarely, Never, I did not have sex in the past 12 months), and how would you rate the level of your sexual attraction to men? (Very high, High, Medium, Low, Very low, None). The same questions were asked regarding woman/women. Composite same-sex and “other”-sex attraction scores were calculated as the mean of the relevant four questions (e.g., for women, the same-sex attraction score was the average score on the four questions relating to women: erotic fantasies with women as the object of fantasy, sex with women, romantic relations with women, and attraction to women; Cronbach’s alpha over the entire sample for same-sex attraction = 0.91 and for “other”-sex attraction = 0.93). Using both same- and “other”-sex attraction scores is grounded in evidence supporting a bi-dimensional continuous conceptualization of sexual orientation (Vrangalova & Savin-Williams, 2012). In addition to completing the sexual orientation questionnaire, participants were asked, in the demographic part of the questionnaire, to mark a sexual orientation category they identified with (exclusively heterosexual, mostly heterosexual, bisexual, mostly homosexual, exclusively homosexual, pansexual, asexual or other). These self-labeled sexual orientation groups differed, on average, in their composite scores on same-sex and “other”-sex attraction (Table 4).

**Table 4:** Mean (SD) of the composite scores of same-sex attraction and “other”-sex attraction in men and women across the five sexual orientation groups

	Exclusively heterosexual^1^	Mostly heterosexual^2^	Bisexual^3^	Mostly Homosexual^4^	Exclusively Homosexual^5^
*Men*					
“Other”-sex attraction	3.67^3,4,5^	3.60^3,4,5^	2.86^1,2,4,5^	0.98^1,2,3,5^	0.24^1,2,3,4^
	(0.52)	(0.52)	(0.92)	(0.69)	(0.31)
Same-sex attraction	0.22^2,3,4,5^	0.90^1,3,4,5^	2.13^1,2,4,5^	3.54^1,2,3,5^	3.90^1,2,3,4^
	(0.34)	(0.61)	(1.00)	(0.59)	(0.25)
*Women*					
“Other”-sex attraction	3.50^3,4,5^	3.56^3,4,5^	3.09^1,2,4,5^	1.24^1,2,3,5^	0.37^1,2,3,4^
	(0.60)	(0.47)	(0.79)	(0.76)	(0.43)
Same-sex attraction	0.46^2,3,4,5^	1.21^1,3,4,5^	2.14^1,2,4,5^	3.31^1,2,3,5^	3.71^1,2,3,4^
	(0.47)	(0.58)	(0.79)	(0.67)	(0.47)

Superscripted numbers represent significant difference (*p* < .01) from the respective sexual orientation group/s within the gender group

The Demographic Questionnaire included items concerning sex at birth (male, female, intersex, other), rearing gender (boy, girl), and adult gender (man, woman, transman, transwoman, transgender, genderqueer, and other), age, place of origin, residency (both current and during childhood), education, and religion. In addition, feminist and queer attitudes were assessed, each by a single item (“Do you hold feminist/queer views?”).

### Data Analysis

Nominal variables and variables with fewer than four values from the demographic questionnaire were analyzed using Chi-square followed by standardized residuals analysis (Sharpe, 2015). We treated as significant, standardized residuals that were larger than 2 or smaller than − 2 (Sharpe, 2015). Ordinal variables obtained from the demographic questionnaire or computed from the answers to the gender and sexuality questionnaires were analyzed using ANOVA and Mann–Whitney *U* test. In the main analysis, we conducted exploratory trend analysis (Bautista, 2008; Tabachnick & Fidell, 2001) for linear and quadratic trends. Significant trends were followed by Tukey post hoc comparisons.

Due to the fact that analyses of large-scale data might produce significant results for even small between-groups differences, Cohen’s *d* was also calculated. All Cohen’s *d* are reported in absolute value. Effect sizes of 0.2, 0.5, and 0.8 were considered small, medium, and large, respectively (Cohen, 1992). In calculating Cohen’s *d,* we weighted the variances according to the proportion of each sexuality group in “Wave 2 of the National Epidemiologic Survey on Alcohol and Related Conditions” (Bostwick, Boyd, Hughs, & McCabe, 2010). We have also adopted Cohen’s (1992) criterion for interpreting the size of significant correlations, treating correlations of 0.1, 0.3, and 0.5 as small, medium, and large, respectively. Because in the present study the relations between sexual orientation and the different aspects of gender identity often included a quadratic element (with the bisexual group at the tipping point), we calculated each correlation between sexual attraction and measures of gender identity three times: once over the entire sample, once over the exclusively heterosexual, mostly heterosexual, and bisexual groups (ExcHet-Bi), and once over the bisexual, mostly homosexual, and exclusively homosexual groups (Bi-ExcHom).

## Results

### Feeling as the Affirmed Gender and Feeling as the “Other” Gender

**Fig. 1:**
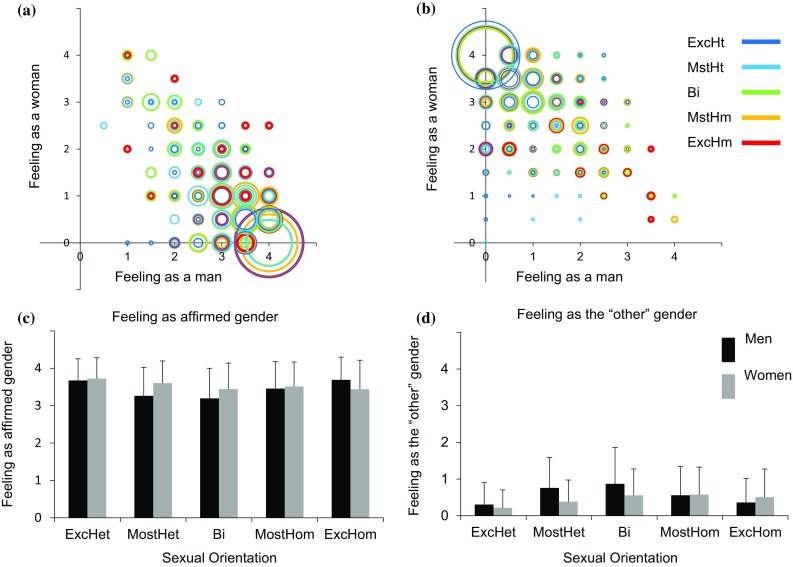
Feeling-as-a-man and feeling-as-a-woman as a function of sexuality. **a**, **b** A scatter plot of feeling-as-a-man (*X*-axis) and feeling-as-a-woman (*Y*-axis) in men (**a**) and women (**b**). Each sexual orientation group is marked in a different color. The size of each circle is proportional to the percent of individuals from a given sexual orientation category with an identical score on the two measures. **c**, **d** The mean and standard deviation of **c** feeling-as-affirmed-gender and **d** feeling-as-the-“other”-gender. *ExcHt* exclusively heterosexual, *MstHt* mostly heterosexual, *Bi* bisexual, *MstHm* mostly homosexual, *ExcHm* exclusively homosexual

**Fig. 2:**
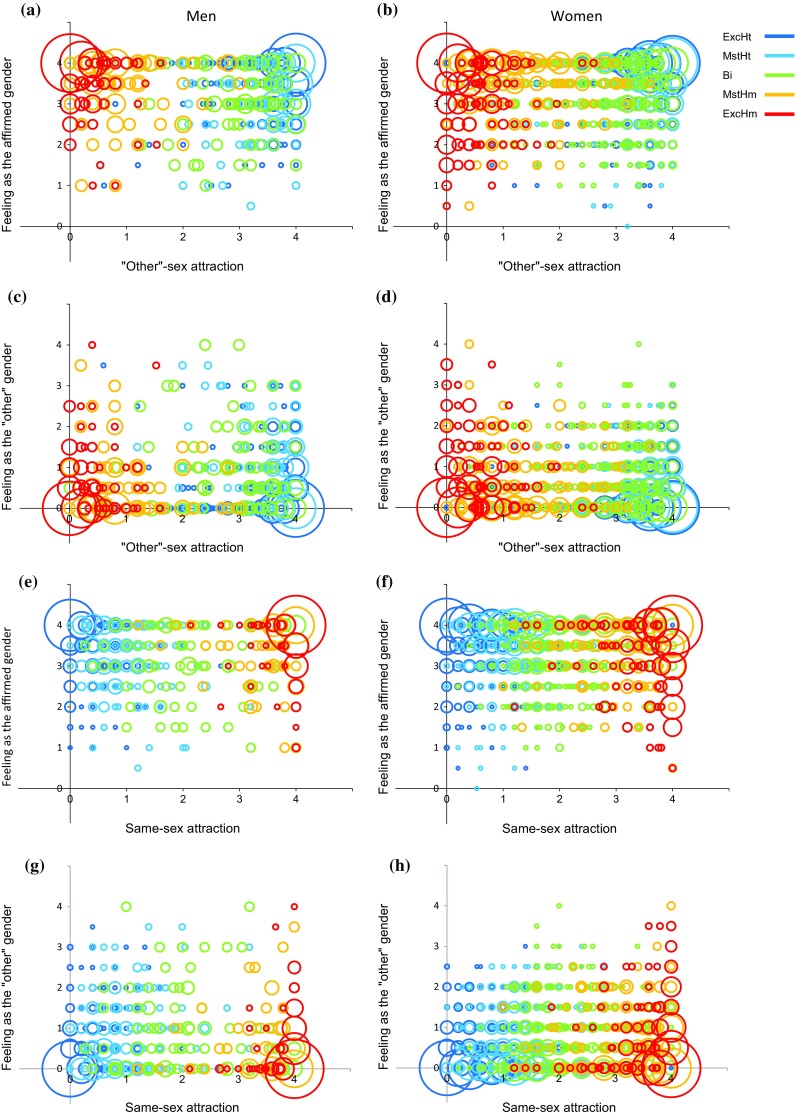
Feeling as the affirmed/”other” gender (*Y*-axis) as a function of same and “other”-sex attraction (*X*-axis) in men and women in the different sexuality groups. (**a**, **b**, **e**, **f**) scatter plots of feeling-as-affirmed-gender and (**c**, **d**, **g**, **h**), scatter plots of feeling-as-“other”-gender as a function of “other”-sex (**a**–**d**) and same-sex (**e**–**h**) attraction. Each sexual orientation group is marked in a different color. The size of each circle is proportional to the percent of individuals from a given sexual orientation category with an identical score on the two measures. *ExcHt* exclusively heterosexual, *MstHt* mostly heterosexual, *B*i bisexual, *MstHm* mostly homosexual, *ExcHm* exclusively homosexual

Figure 1a–d presents the scores (1a, b) and mean and SD (1c, d) of feeling-as-a-man (*X*-axis) and feeling-as-a-woman (*Y*-axis) in men and women as a function of their self-labeled sexual orientation category. As we have previously found (Joel et al., 2013), the perception of gender identity of both women and men was highly variable and was mainly related to one’s affirmed gender rather than to one’s sexual orientation. This is also evident in Fig. 2 which depicts on the *Y*-axis the scores of feeling-as-affirmed-gender (2a, b, e, f) and feeling-as-“other”-gender (2c, d, g, h) as a function of “other”-sex (2a–d) and same-sex (2e–h) attraction, in men (2a, c, e, g) and women (2b ,d, f, h) belonging to the five sexual orientation categories. As can be seen, self-reported gender identity is similarly scattered along the sexuality scale. This is particularly evident for the exclusively heterosexual and exclusively homosexual groups, because they show similar distributions along the sexual attraction scale.

At the group level, the present study reveals that the relations between sexual orientation and the perception of gender identity were different in men and women (Fig. 1c, d). In men, the relations between feeling-as-affirmed-gender and sexual orientation were dominated by a U-shaped trend (for the full results of the trends analysis see Table 5), being highest at both ends of the sexual orientation continuum (i.e., the exclusively heterosexual and exclusively homosexual groups) and lowest in its center (i.e., the bisexual group) (for the results of the post hoc comparisons between the different sexual orientation groups within each gender, see Table 6). The relations between feeling-as-the-”other”-gender and sexual orientation had an inverted-U shape, being lowest in the exclusively heterosexual and exclusively homosexual groups, and highest in the bisexual group (Tables 5, 6). In women, these relations were a combination of linear and quadratic relations, such that there was a gradual change from exclusively heterosexual to bisexual with no further changes from bisexual to exclusively homosexual (a pattern we call here “mostly linear”). This was true for both feeling-as-affirmed-gender and feeling-as-the-”other”-gender (Tables 5, 6).

**Table 5:** Trend analysis for the Multi-GIQ variables in men and women

	Men		Women	
	Linear trend	Quadratic trend	Linear trend	Quadratic trend
Feeling as affirmed gender	ns	*F*(1, 4745) = 77.86, *p* < .001	*F*(1, 4745) = 53.80, *p* < .001	*F*(1, 4745) = 10.04, *p* = .002
Feeling as the “other” gender	ns	*F*(1, 4746) = 83.95, *p* < .001	*F*(1, 4746) = 70.27, *p* < .001	*F*(1, 4746) = 36.59, *p* < .001
Feeling as both genders	ns	*F*(1, 4744) = 72.02, *p* < .001	*F*(1, 4744) = 109.19, *p* < .001	*F*(1, 4744) = 80.47, *p* < .001
Feeling as neither gender	ns	ns	*F*(1,4727) = 35.87, *p* < .001	ns
Satisfaction with one’s affirmed gender	ns	*F*(1, 4695) = 69.75, *p* < .001	ns	*F*(1,4695) = 34.66, *p* < .001
Wish to be the “other” gender	ns	*F*(1, 4304) = 87.23, *p* < . 001	*F*(1,4304) = 21.14, *p* < .001	*F*(1, 4304) = 55.71, *p* < .001
Dislike of one’s sexed body	ns	*F*(1, 4640) = 46.88, *p* < 0.001	*F*(1, 4640) = 21.99, *p* < .001	ns
Wish to have the body of the “other” sex	*F*(1,4732) = 22.66, *p* < .001	*F*(1,4732) = 134.55, *p* < .001	*F*(1, 4732) = 77.38, *p* < .001	*F*(1, 4732) = 50.73, *p* < .001
Wearing the clothes of the other sex	ns	*F*(1, 4736) = 75.19, *p* < .001	*F*(1,4736) = 387.40, *p* < .001	*n*s
Shopping in a department labeled for your sex	*F*(1, 4740) = 18.65, *p* < .001	*F*(1, 4740) = 9.09, *p* = .003	*F*(1, 4740) = 577.65, *p* < .001	*F*(1, 4740) = 26.47, *p* < .001

**Table 6:** Cohen’s *d* for significant comparisons between sexual orientation categories

Variable	ExcHet	MostHet	Bi	MostHom	ExcHom
*Feeling as affirmed gender*					
ExcHet	–	0.54	0.59	ns	ns
MostHet	0.21*	–	ns	ns	− 0.67
Bi	0.41	0.24	–	ns	− 0.70
MostHom	0.33	ns	ns	–	ns
ExcHom	0.37	0.22*	ns	ns	–
*Feeling as “other” gender*					
ExcHet	–	− 0.55	− 0.57	ns	ns
MostHet	− 0.28	–	ns	ns	0.55
Bi	− 0.47	− 0.25	–	ns	0.58
MostHom	− 0.49	− 0.27*	ns	–	ns
ExcHom	− 0.38	ns	ns	ns	–
*Feeling as both genders*					
ExcHet	–	− 0.65	− 0.70	0.46*	ns
MostHet	− 0.42	–	ns	ns	0.49
Bi	− 0.67	− 0.31	–	ns	0.54
MostHom	− 0.63	− 0.26	ns	–	ns
ExcHom	− 0.52	ns	ns	ns	–
*Feeling as neither gender* ^a^					
ExcHet	–				
MostHet	− 0.30	–			
Bi	− 0.48	− 0.20	–		
MostHom	− 0.40	ns	ns	–	
ExcHom	− 0.33	ns	ns	ns	–
*Satisfaction with affirmed gender*					
ExcHet		0.59	0.64	ns	ns
MostHet	0.29		ns	ns	− 0.70
Bi	0.35	ns		ns	− 0.75
MostHom	ns	ns	ns		ns
ExcHom	ns	− 0.25*	− 0.31	ns	
*Wish to be the “other” gender*					
ExcHet		− 0.60	− 0.72	ns	ns
MostHet	− 0.36		ns	ns	0.67
Bi	− 0.49	ns		ns	0.78
MostHom	− 0.45	ns	ns		ns
ExcHom	− 0.26	ns	ns	ns	
*Dislike of one’s sexed body*					
ExcHet		− 0.48	− 0.59	ns	ns
MostHet	ns		ns	ns	0.56
Bi	− 0.24	ns		0.59*	0.66
MostHom	ns	ns	ns		ns
ExcHom	− 0.27	ns	ns	ns	
*Wish to have the body of the “other” sex*					
ExcHet		− 0.69	− 0.71	ns	ns
MostHet	− 0.30		ns	0.60	0.93
Bi	− 0.60	− 0.35		0.66	0.89
MostHom	− 0.51	− 0.26	ns		ns
ExcHom	− 0.40	ns	ns	ns	
*Wearing the clothes of the “other” sex*					
ExcHet		0.51	0.69	ns	ns
MostHet	0.40		ns	ns	− 0.57
Bi	0.60	0.21		ns	− 0.76
MostHom	0.72	0.36	ns		ns
ExcHom	1.02	0.72	0.57	0.40	
*Shopping in a department labeled for your sex*					
ExcHet		ns	ns	ns	ns
MostHet	0.32		ns	ns	− 0.47
Bi	0.53	0.24		ns	ns
MostHom	0.71	0.47	0.27		ns
ExcHom	1.06	0.92	0.80	0.54	

Values above and under the diagonal refer to the men and women’s groups, respectively

Cohen’s *d* between any two groups was calculated by deducing the mean of the group to the right from the mean of the group to the left

^a^In the men’s group trend analysis for feeling as neither gender was insignificant and therefore post hoc comparisons were not calculated

Unless marked otherwise, *p* < .001

*ExcHet* exclusively heterosexual, *MostHet* mostly heterosexual, *Bi* bisexual, *MostHom* mostly homosexual, *ExcHom* exclusively homosexual

*p < .01

**Fig. 3:**
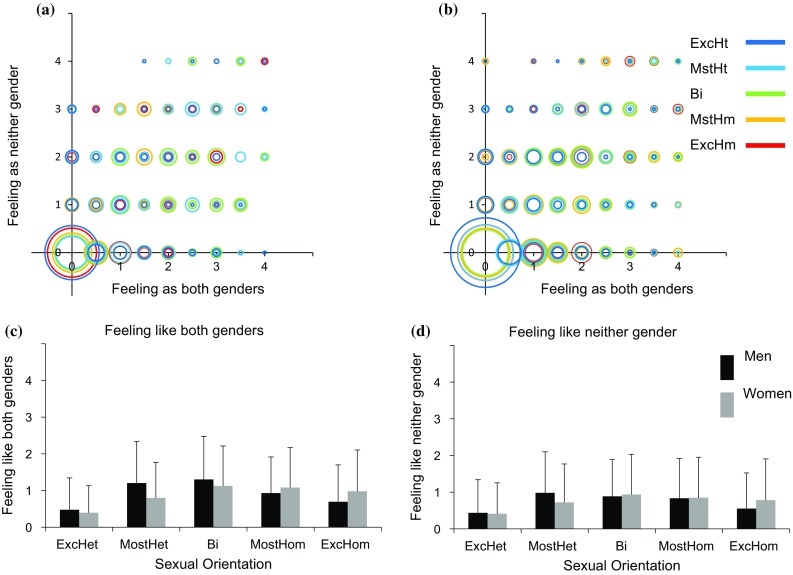
Feeling-as-both-genders (*X*-axis) and feeling-as-neither-gender (*Y*-axis) as a function of sexuality. **a**, **b** A scatter plot of feeling-as-both-genders and feeling-as-neither-gender in men (**a**) and women (**b**). Each sexual orientation group is marked in a different color. The size of each circle is proportional to the percent of individuals from a given sexual orientation category with an identical score on the two measures. **c**, **d** The mean and standard deviation of **c** feeling-as-both-genders and **d** feeling-as-neither-gender. *ExcHt* exclusively heterosexual, *MstHt* mostly heterosexual, *Bi* bisexual, *MstHm* mostly homosexual, *ExcHm* exclusively homosexual

Similarly, the relations between feeling-as-both-genders (Fig. 3c; Tables 5, 6) and sexual orientation were quadratic in men, and mostly linear in women. The relations between women’s feeling-as-neither-gender and sexual orientation were linear, whereas for men, trend analysis for feeling-as-neither-gender was not significant (Fig. 3d; Tables 5, 6).

Table 7 presents the correlations between the composite variables same- and “other”-sex attraction and feeling-as-affirmed-gender, feeling-as-the-”other”-gender, feeling-as-both-genders, and feeling-as-neither-gender for men and for women, separately, when these correlations were calculated over the entire sample, as well as when they were calculated over the exclusively heterosexual, mostly heterosexual, and bisexual groups (the ExcHet-Bi sub-sample), or over the bisexual, mostly homosexual, and exclusively homosexual groups (the Bi-ExcHom sub-sample). The correlations were small, with none (out of 48) exceeding 0.3, and in the direction expected according to the results of the trends analyses. Specifically, in men, the sign of the correlations between measures of sexual attraction and the different aspects of the perception of gender identity was opposite in the ExcHet-Bi and the Bi-ExcHom sub-samples, whereas in women, only 1 out of the 17 significant correlations was in the Bi-ExcHom sub-sample.

**Table 7:** Correlations between the composite variables same- and “other”-sex attraction and the different aspects of gender identity, over the entire gender group (all, top numbers), the exclusively heterosexual, mostly heterosexual and bisexual groups (ExcHet-Bi, middle numbers) and the bisexual, mostly homosexual and exclusively homosexual groups (Bi-ExcHom, bottom numbers)

	Feel as affirmed gender	Feel as “other” gender	Feel as both genders	Feel as neither gender	Satisfied with my gender	Wish to be the “other” gender	Dislike of the sexed body	Wish to have the body of the “other” sex	Wearing the clothes of the “other” sex	Shopping in a department labeled for your sex
*Men*										
“Other”-sex attraction										
All	− .02	− .01	− .04	− .01	− .03	.02	.04	.10*	− .04	− .10*
ExcHet-Bi	.23*	− .19*	− .15*	− .08	.20*	− .16*	− .16*	− .14*^#^	.08^#^	.05
Bi-ExcHom	− .30*	.24*	.22*	.18*	− .25*	.30*	.26*	.39*	− .36*	− .17*
Same-sex attraction										
All	− .01	.07	.11*	.06	.00	.06	.01	− .00	− .05	.10*
ExcHet-Bi	− .26*	.29*	.30*	.16*	− .23*	.32*	.25*	.33*^#^	− .26*^#^	− .03
Bi-ExcHom	.28*	− .21*	− .19*	− .15*	.25*	− .26*	− .26*	− .34*	.30*	.14*
*Women*										
“Other”-sex Attraction										
All	.13*	− .11*	− .13*	− .09*	− .00	− .04	− .08*	− .10*	.24*	.36*
ExcHet-Bi	.14*	− .07*	− .10*	− .11*	.10*	− .03	− .07*	− .07*^#^	.04^#^	.12*^#^
Bi-ExcHom	.01	.02	.04	.05	− .12*	.08*	− .04	.06	.20*	.32*
Same-sex attraction										
All	− .14*	.19*	.24*	.13*	− .03	.15*	.10*	.22*	− .29*	− .35*
ExcHet-Bi	− .14*	.21*	.27*	.17*	− .11*	.19*	.11*	.25*^#^	− .19*^#^	− .19*^#^
Bi-ExcHom	.03	− .01	− .03	− .09*	.16*	− .05	.01	− .01	− .15*	− .25*

*ExcHet* exclusively heterosexual, *Bi* bisexual, *ExcHom* exclusively homosexual

*Correlation (Pearson’s r) is significant at the .01 level (two-tailed)

^#^Within the gender group, same-sex attraction is significantly (*p* < .01) more related to the variable than “other”-sex attraction

### Additional Aspects of Gender Identity

**Fig. 4:**
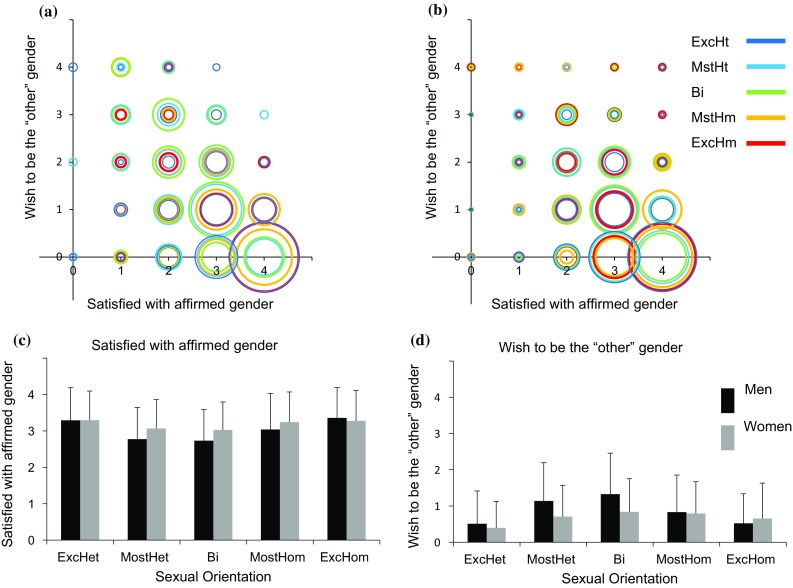
Satisfied-with-affirmed-gender and wish-to-be-the-“other”-gender as a function of sexuality. **a**, **b** A scatter plot of satisfied-with-affirmed-gender (*X*-axis) and wish-to-be-the-“other”-gender (*Y*-axis) in men (**a**) and women (**b**). Each sexual orientation group is marked in a different color. The size of each circle is proportional to the percent of individuals from a given sexual orientation category with an identical score on the two measures. **c**, **d** The mean and standard deviation of **c** satisfied-with-affirmed-gender and **d** wish-to-be-the-“other”-gender. *ExcHt* exclusively heterosexual, *MstHt* mostly heterosexual, *Bi* bisexual, *MstHm* mostly homosexual, *ExcHm* exclusively homosexual

**Fig. 5:**
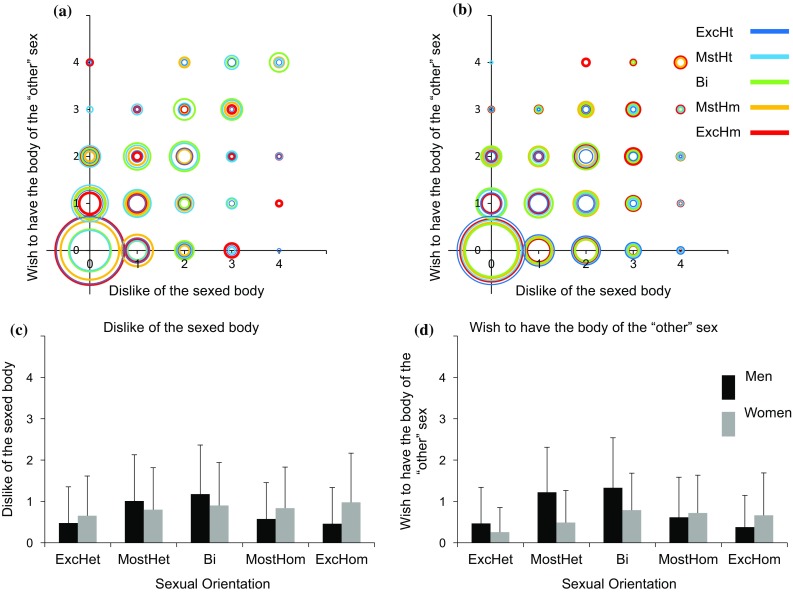
Dislike-of-the-sexed-body and wish-to-with-to-have-the-body-of-the-“other”-sex as a function of sexuality. **a**, **b** A scatter plot of dislike-of-the-sexed-body (*X*-axis) and wish-to-with-to-have-the-body-of-the-“other”-sex (*Y*-axis) in men (**a**) and women (**b**). Each sexual orientation group is marked in a different color. The size of each circle is proportional to the percent of individuals from a given sexual orientation category with an identical score on the two measures. **c**, **d** The mean and standard deviation of **c** dislike-of-the-sexed body and **d** ish-to-with-to-have-the-body-of-the-“other”-sex. *ExcHt* exclusively heterosexual, *MstHt* mostly heterosexual, *Bi* bisexual, *MstHm* mostly homosexual, *ExcHm* exclusively homosexual

**Fig. 6:**
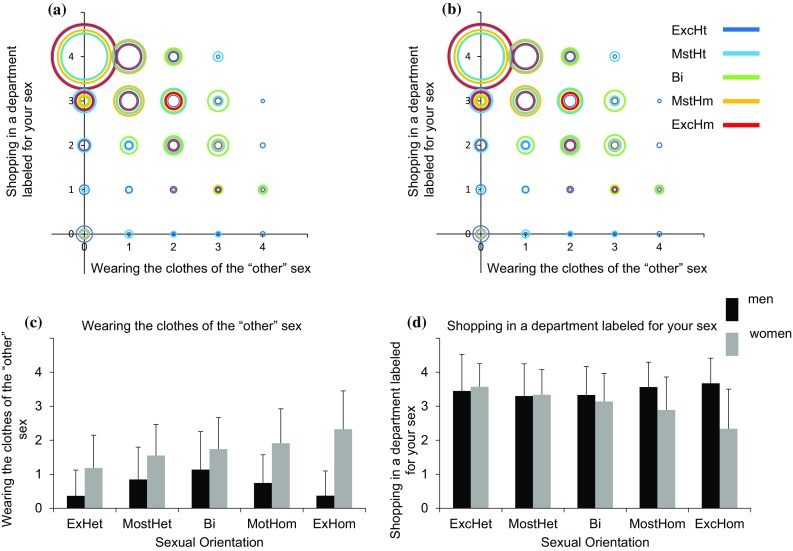
Wearing-the-clothes-of-the-“other”-sex and shopping-in-a-department-labeled-for-your-sex as a function of sexuality. **a**, **b** A scatter plot of wearing-the-clothes-of-the-“other”-sex (*X*-axis) and shopping-in-a-department-labeled-for-your-sex (*Y*-axis) in men (**a**) and women (**b**). Each sexual orientation group is marked in a different color. The size of each circle is proportional to the percent of individuals from a given sexual orientation category with an identical score on the two measures. **c**, **d** The mean and standard deviation of **c** wearing-the-clothes-of-the-“other”-sex and **d** shopping-in-a-department-labeled-for-your-sex. *ExcHt* exclusively heterosexual, *MstHt* mostly heterosexual, *Bi* bisexual, *MstHm* mostly homosexual, *ExcHm* exclusively homosexual

The results of the analyses of the relations between sexuality and satisfaction-with-one’s-affirmed-gender (Fig. 4c), the wish-to-be-the-“other”-gender (Fig. 4d), dislike-of-one’s-sexed-body (Fig. 5c), the wish-to-have-the-body-of-the-“other”-sex (Fig. 5d), wearing-the-clothes-of-the-“other”-sex (Fig. 6c), and shopping-in-a-department-labeled-for-your-sex (Fig. 6d), are shown in Tables 5, 6, and 7. In general, on all these variables, the responses of women and men were highly variable and overlapping, and were related mostly to affirmed gender, and only weakly to sexual orientation. The relations between these variables and sexuality followed a similar pattern to the one described above, that is, a quadratic trend in men, and a combination of linear and quadratic trends in women (see Table 5 for the full results). In addition, the correlations of the different variables with same- and “other”-sex attraction were small, with only 8 (out of 72) exceeding 0.3, and in the direction expected according to the results of the trends analyses. Finally, in only five cases, the correlations of the different variables with same-sex attraction were significantly larger (in absolute terms) from their correlations with “other”-sex attraction (see Table 7 for the five statistically significant differences).

### “Binary” Versus “Non-binary” Gender experiences

As evident in Figs. 1, 2, 3, 4, and 5, many participants had what may be termed “queer” feelings, such as feeling both as a man and as a woman (38%) or as neither (35%), wishing to be the “other” gender (38%), or wishing to have the body of the “other” sex (35%). On the other hand, there were many individuals who had what may be viewed as a “binary” response pattern, such as always feeling as a man and never feeling as a woman. Table 8 presents for each of the gender and sexual orientation groups the percent of participants with a completely binary response pattern over aspects of gender identity relevant to this binary–non-binary distinction. Thus, a completely binary response pattern for a woman would be: always feeling like a woman, never feeling like a man, never feeling as both genders, never feeling as neither gender, never wishing to be the “other” gender, and never wishing to have the body of the “other” sex. The items, satisfied-with-my-own-gender, dislike-of-one’s-sexed-body, wearing-clothes-of-the-“other”-sex, and shopping-in-a-department-labeled-for-one’s-sex, were not included because responses different from Never or Always on these items may be attributed to other factors (e.g., body size not typical to one’s sex, wearing the boyfriend’s T-shirt). In all cases, the responses of participants with a completely binary response pattern were in line with their self-identified gender. The percent of binary individuals ranged between 8.8 and 40%, depending mostly on sexual orientation, with the highest percentage of binary individuals found in the exclusively heterosexual and exclusively homosexual groups, which did not significantly differ (*p* = .80*)*, and lower percentage in the mostly heterosexual, bisexual, and mostly homosexual groups (for the results of the logistic regression see Table S3 in Supplementary materials).

**Table 8:** Percent of participants with a binary response pattern in each group

	ExcHet (%)	MstHet (%)	Bi (%)	MstHom (%)	ExcHom (%)
Men	38.0	9.1	11.8	16.7	33.0
Women	41.8	20.9	13.5	16.0	28.1

*ExcHet* exclusively heterosexual, *MstHet* mostly heterosexual, *Bi* bisexual, *MstHom* mostly homosexual, *ExcHom* exclusively homosexual

## Discussion

The main findings of the present study are that cisgender individuals may show a wide range of gender experiences that deviate from the expected binary (Figs. 1a, b, 2, 3a, b, 4a, b, 5a, b, 6a, b) and that variability in the different aspects of gender identity is only weakly related to sexual orientation.

Before further summarizing the results, we would like to describe several limitations of our study. The study used a convenience sample achieved by recruiting participants online, through mailing lists, Internet posts and snowballing, and no means were taken to achieve random sampling. Indeed, the percentage of non-heterosexuals in the present sample (63.23%) is much higher than the average percentage reported in a recent review of sexual-identity distributions (Bailey et al., 2016) (~ 7% in men and ~ 13% in women). Moreover, the fact that the invitation to participate in the study revealed its major aims and was published also in LGBT-focused venues, may have biased our sample toward more feminist or queer-minded participants. We included only participants from countries where English was a formal language; to assess participants’ gender perception, we used the Multi-GIQ, which, being closed-ended and based on a binary conception of gender, may limit the representation of identities that transcend this conception in additional ways; and last, we relied on self-reports, which could have been influenced by impression management. In view of these limitations, we view our results more as reflecting the range of experiences in cisgender individuals in western, developed countries, than as providing exact estimates of the proportion of each experience in cisgender populations around the world. Future research will benefit from using large-scale, representative samples from more versatile cultural climates, using more open-ended explicit assessment of gender identity or rather implicit measures of gender identity (e.g., Greenwald, McGhee, & Schwartz, 1998).

Our analysis of different aspects of gender identity revealed that the majority of the participants experienced themselves much more as their affirmed gender than as the “other” gender, showed high levels of satisfaction with their affirmed gender and low levels of dissatisfaction with their sexed body, as well as low levels of desire to be the “other” gender or to have the body of the “other” sex, and high levels of compliance with expected gender performance in terms of clothing. Yet, as in Joel et al. (2013), many cisgender individuals had quite “queer” feelings, with 38% of the participants in the present sample feeling to some degree as the “other” gender, 39% wishing to some extent they were the “other” gender, and 35% wishing at least rarely that they had the body of the “other” sex. On the other hand, the percentage of individuals who were completely “binary” in their responses across all aspects of gender identity was 25.50%.

The present study also replicates and extends Joel et al.’s (2013) finding that variability in gender identity is only weakly related to sexual orientation. This was best evident in the low correlations between the different aspects of gender identity and same- and “other”-sex attraction, both when these were calculated over all women or all men, and when they were calculated over only half of the sexual orientation continuum (i.e., on the exclusively heterosexual, mostly heterosexual, and bisexual groups or on the bisexual, mostly homosexual, and exclusively homosexual groups). Of the 120 correlations computed, only eight exceeded 0.30 (none exceeded 0.40). There were no consistent differences between correlations with same-sex attraction versus “other”-sex attraction, except that, in both women and men, but only in the more heterosexual half of the sexual orientation continuum, same-sex attraction was more related than “other”-sex attraction to the wish-to-have-the-body-of-the-”other”-sex, wearing-the-clothes-of-the-”other”-sex, and shopping-in-a-department-labeled-for-your-sex (the latter was significant only in the women’s group). The weak relation between sexual orientation and gender identity was also evident in the effect size of the differences between the five sexual orientation groups within each gender. Thus, of the 190 pair-wise comparisons, 50% were nonsignificant, 13.7% were between 0.20 and 0.35, 23.7% were between 0.35–0.65 and 12.6% were larger than 0.65 (the largest was 1.06). Moreover, the differences between the different sexual orientation groups within each gender were small compared to the differences between the two genders (e.g., Cohen’s *d* of the difference between women and men on feeling-as-a-woman and on feeling-as-a-man was 4.5 and 4.8, respectively, compared with the largest difference within each gender on these variables which was 0.70).

At first inspection, the finding that the correlations between measures of gender identity and of sexual attraction in cisgender individuals are small seems to contradict previous studies that reported higher gender nonconformity in non-heterosexual adults (e.g., Bailey, 2003; Lippa, 2005a, 2005b, 2008; Peters et al., 2007; Rahman et al., 2003; Rieger et al., 2008, 2010; Rule et al., 2008; Su, Rounds, & Armstrong, 2009). The present study differs, however, from previous studies in two respects. The first is that the present study focused on gender identity and therefore assessed only a few of the many different aspects of gender nonconformity that were assessed in previous studies (such as occupational and recreational interests, patterns of movement, speech and physical appearance, cognitive abilities, and personality traits). Indeed, in line with our finding, a study that assessed gender identity in males reporting gender dysphoria, did not find significant differences between the different sexual orientation groups (Deogracias et al., 2007). The second is that the present study used a five-level categorical measure of sexual orientation as well as a continuous measure of sexual attraction, whereas most previous studies only compared groups of heterosexual and homosexual individuals (see Bailey et al., 2016 for a summary of the relevant literature). When we used our data to compare heterosexual individuals (that is, the exclusively heterosexual and mostly heterosexual groups of the present study) with non-heterosexual individuals (that is, the bisexual, mostly homosexual, and exclusively homosexual groups of the present study) on the gender nonconformity-related variables that were included in the present study (namely, feeling-as-the-“other”-gender, wish-to-be-the-“other”-gender, wish-to-have-the-body-of-the-“other”-sex, wearing-the-clothes-of-the-“other”-sex, and shopping-in-a-department-labeled-for-your-sex), significant differences were found (Cohen’s *d* ranged from 0.03 to 0.58), with the non-heterosexual group showing on average more gender nonconformity, as previously reported. The presence of such differences, found here and in numerous other studies, demonstrates that at the group level, non-heterosexual sexuality is linked with gender nonconformity, but that a more nuanced analysis reveals that the relation between sexuality and gender identity, is weak.

The present study further reveals that the relations between the categories of sexual orientation and the different aspects of gender identity were gender-specific, being mostly U shaped or inverted-U shaped in men and mostly linear in women. Thus, in women, feeling-as-a-woman was highest in the exclusively heterosexual group, somewhat lower in the mostly heterosexual group, and lowest in the bisexual, mostly homosexual, and exclusively homosexual groups, which did not differ, and the reverse was true for feeling-as-a-man (i.e., lowest in the exclusively heterosexual group and highest in the bisexual, mostly homosexual, and exclusively homosexual groups). In men, feeling-as-a-man was highest at both ends of the sexual orientation continuum (i.e., the exclusively heterosexual and exclusively homosexual groups) and lowest at its center (i.e., the bisexual group), and the reverse was true for feeling-as-a-woman. Similar U- or inverse-U-shaped relations in men, and mostly linear relations in women were evident also for most of the other aspects of gender identity. The different relations between sexual orientation and the different aspects of gender identity in women and men were also evident in the correlations between sexual attraction and measures of gender identity. Thus, for all measures, in men, the sign of the correlations was always opposite in the ExcHet-Bi and the Bi-ExcHom sub-samples, whereas in women, there were very few significant correlations in the Bi-ExcHom sub-sample.

The present findings conflict with the common postulation of direct relations between biological sex, gender identity, and sexual orientation in two major aspects, which are clearly evident in Fig. 1. First, while scientific discourse usually perceives gender identity as a clear-cut, binary personality structure, our data reveal large variability in individuals’ gender identity with about a third feeling at least to some degree as the “other” gender. Second, and out of line with the idea that an “atypical” sexual orientation would entail an “atypical” gender identity, variability in gender identity was evident throughout the sexual attraction continuum, with an almost complete overlap between heterosexuals and non-heterosexuals in the range of scores on the different measures of gender identity. Moreover, even at the group level, only some non-heterosexual groups were significantly different from the exclusively heterosexual group. In fact, the finding that the group of exclusively homosexual men was not significantly different from the group of exclusively heterosexual men on any of the measures of gender identity is particularly in conflict with views strongly linking sexual orientation and gender identity. Our findings are in agreement, however, with the view that sexual orientation and gender identity are mostly distinct constructs (Burman, 2005; Connell, 1985; Jackson, 2006; Morgan, 2013; Shively & De Cecco, 1977; Striepe & Tolman, 2003; Vanwesenbeeck, 2009).

There are several possible explanations for the weak relations that were found between sexual orientation and gender identity. One is a correlation between these variables and a third variable, such as flexibility or openness. For example, synthesizing eight studies, Lippa (2005a, 2005b, 2008) found medium-sized Cohen *d*’s (0.42 and 0.47 for men and women, respectively) for “openness to experience” in favor of gay men and lesbian women compared to heterosexual men and women, and a recent study reported that children who feel similarity to the “other” gender as well as to their own gender may enjoy added flexibility in their social lives (Martin et al., 2017).

Another possible account of the weak relations between sexual orientation and gender identity revolves around the scripted nature of sexual interactions and their role in the construction of gender (MacKinnon, 1989). Several theoreticians have claimed that sexual interaction is one of the domains where men and women feel most pressured to enact gender roles (Coward, 1985; Rohlinger, 2002; Sanchez, Crocker, & Boike, 2005). Following this line of thought, it is possible that negotiating sexuality within a same-sex interaction allows one partner at a time to enact his/her affirmed gender role, while the other partner occupies and explores a position deemed by the gender norms as belonging to the “other” gender. This might explain why more same-sex experiences might entail more experience with non-traditional gender roles and thus less dichotomous gender identification and performance.

The different relations between sexuality and gender identity in men and women may be related to the different attitudes toward same-sex sexual relations of women and men in western society. Specifically, same-sex relations are less acceptable for men compared to women and are often considered un-masculine (Herek, 1986, 2000; Pew Center, 2013). This cultural difference may explain, at least in part, why even low levels of same-sex sexuality were associated with less binary gender identity in men compared to women. Thus, mostly heterosexual and bisexual men were on average less binary than women in the respective sexuality groups. This account does not explain, however, the observation that exclusively homosexual men scored on average identically to exclusively heterosexual men.

The similar gender identity in exclusively heterosexual and exclusively homosexual men, but not women, may be accounted for by the role of sexual interactions in the construction of gender in terms of power relations. In western cultures, being male and masculinity have a higher value than being female and femininity. Research has consistently shown that many gay men tend to value masculinity and perceive negatively those who appear effeminate to them (Bergling, 2001; Sánchez, 2016). Being subjected to the same cultural messages regarding masculinity and effeminacy as heterosexual men (Wilson et al., 2010), many gay men come to define masculinity and femininity as heterosexual men do (Sánchez, Greenberg, Liu, & Vilain, 2009). The high value gay men place in masculinity is evident in higher rates of masculine self-presentation within romantic and sexual contexts in comparison with heterosexuals as well as in a desire for masculine partners (Asencio, 2011; Bailey, Kim, Hills, & Linsenmeier, 1997; Bartholome, Tewksbury, & Bruzzone, 2000; Bianchi et al., 2010; Deaux & Hanna, 1984; Downing & Schrimshaw, 2014; Gudelunas, 2005; Laner & Kamel, 1978; Logan, 2010; Lumby, 1978; Malebranche, Fields, Bryant, & Harper, 2007; Sánchez & Vilain, 2012; Sánchez, Westefeld, Liu, & Vilain, 2010; Ward, 2008). Further evidence indicates that gay men show bias against femininity in general (Bailey et al., 1997; Skidmore, Linsenmeier, & Bailey, 2006). Together, these pro-masculine and anti-feminine positions of homosexual men might make cross-gender identification less desirable and less likely. In contrast, for mostly heterosexual, bisexual, and mostly homosexual men, being in romantic/sexual relations with both men and women may make exploring different gender roles likely and even desirable, while still allowing some degree of freedom from the stigma faced by homosexuals.

### Conclusions

Our findings replicate previous results that even participants who self-identify in “normative” ways (i.e., female-woman, male-man) may experience themselves also as the “other” gender or wish to be the “other” gender (Joel et al., 2013). This finding is important because it highlights the fact that gender identities do not conform to narrowly defined dichotomous framings and suggests that identification with the “other” gender or wish to be the “other” gender or to have the body of the “other” sex are not necessarily a sign of gender dysphoria. Thus, our findings may normalize diversity in an area usually thought of as homogenous, and by doing so help represent queer and transgender identities as belonging on the same gender grid as cisgender identities rather than as distinct phenomena.

More generally, our study adds to a growing body of literature that challenges dichotomous conventions within the science of gender and sexuality (for a recent review, see Hyde, Bigler, Joel, Tate, & van Anders, in press). Our results undermine the tight link assumed to exist between sexual and gender identities, and instead posit these identities as distinct constructs. Replacing the dichotomous view with a more flexible and fluid view of gender and sexuality will accommodate the experiences of cisgender and transgender individuals and enable many to express their gender and sexuality without having to be at risk of harmful consequences.

### Electronic supplementary material

Below is the link to the electronic supplementary material.

Supplementary material 1 (DOCX 20 kb)

## Appendix

### The Multi-Gender Identity Questionnaire

1. In the past 12 months, have you felt satisfied being a woman?

Always, Often, Sometimes, Rarely, Never, Not relevant

2. In the past 12 months, have you felt satisfied being a man?

Always, Often, Sometimes, Rarely, Never, Not relevant

3. In the past 12 months, have you thought of yourself as a woman?

Always, Often, Sometimes, Rarely, Never

4. In the past 12 months, have you thought of yourself as a man?

Always, Often, Sometimes, Rarely, Never

5. In the past 12 months, have you felt that you have to work at being a woman?

Always, Often, Sometimes, Rarely, Never, Not relevant

6. In the past 12 months, have you felt that you have to work at being a man?

Always, Often, Sometimes, Rarely, Never, Not relevant

7. In the past 12 months, have you felt pressured by others to be a “proper” woman?

Always, Often, Sometimes, Rarely, Never, Not relevant

8. In the past 12 months, have you felt pressured by others to be a “proper” man?

Always, Often, Sometimes, Rarely, Never, Not relevant

9. In the past 12 months, have you felt that you were a “real” woman?

Always, Often, Sometimes, Rarely, Never

10. In the past 12 months, have you felt that you were a “real” man?

Always, Often, Sometimes, Rarely, Never

11. In the past 12 months, when you went into a department store to buy yourself clothing, did you shop in a department labeled for your sex?

Always, Often, Sometimes, Rarely, Never

12. In the past 12 months, have you worn the clothes of the other sex?

Always, Often, Sometimes, Rarely, Never

13. In the past 12 months, have you felt more like a man than like a woman?

Always, Often, Sometimes, Rarely, Never

14. In the past 12 months, have you felt more like a woman than like a man?

Always, Often, Sometimes, Rarely, Never

15. In the past 12 months, have you sometimes felt like a man and sometimes like a woman?

Always, Often, Sometimes, Rarely, Never

16. In the past 12 months, have you felt somewhere in between a woman and a man?

Always, Often, Sometimes, Rarely, Never

17. In the past 12 months, have there been times when you’ve felt that you are neither a man nor a woman?

Always, Often, Sometimes, Rarely, Never

18. In the past 12 months, have you felt that it is/it would be better for you to live as a man than as a woman?

Always, Often, Sometimes, Rarely, Never

19. In the past 12 months, have you felt that it is/it would be better for you to live as a woman than as a man?

Always, Often, Sometimes, Rarely, Never

20. In the past 12 months, have you had the wish or desire to be a man?

Always, Often, Sometimes, Rarely, Never, Not relevant

21. In the past 12 months, have you had the wish or desire to be a woman?

Always, Often, Sometimes, Rarely, Never, Not relevant

22. In the past 12 months, have you disliked your body because of its female form?

Always, Often, Sometimes, Rarely, Never, Not relevant

23. In the past 12 months, have you disliked your body because of its male form?

Always, Often, Sometimes, Rarely, Never, Not relevant

24. In the past 12 months, have you wished you had the body of the “other” sex?

Always, Often, Sometimes, Rarely, Never

## Footnotes

1 Many researchers distinguish between *sex* and *gender*—the biological and the social-cultural aspects, respectively, of being male or female. Since the introduction of these terms by Unger (1979), it has become clear that the distinction between sex and gender is not as clear-cut as originally postulated, and that each of these terms has several different meanings (for a recent review, see Jordan-Young & Rumiati, 2012). Here we use *sex* to refer to the category assigned to an infant at birth, typically female or male, and *gender* to refer to the social category one identifies with, most often, woman or man. We use the term “other” gender to refer to the other gender between these two, but use quotation marks around other to remind ourselves and the reader that man and woman are not the only possible categories. For a similar reason, we use “other” sex.
