# Supplementary material for: An Exploration of the Relations Between Self-Reported Gender Identity and Sexual Orientation in an Online Sample of Cisgender Individuals
Source: Arch Sex Behav. 2018 Jul 3;47(8):2407–26. doi: 10.1007/s10508-018-1239-y (PMC6245116; doi:10.1007/s10508-018-1239-y)
Supplement: Supplementary file 1 — Supplementary material 1 (DOCX 20 kb) [file 10508_2018_1239_MOESM1_ESM.docx]

|  | Table 1S. Results of Chi-Square Analysis of Current Living Area, Childhood Living Area, and Feminist Views for Each Sexual Orientation Group in Men and Women | | | | | | | | |  |  |  |  |  |  |
| --- | --- | --- | --- | --- | --- | --- | --- | --- | --- | --- | --- | --- | --- | --- | --- |
|  |  | |  | Exclusively Heterosexual | Mostly Heterosexual | Bisexual | Mostly Homosexual | Exclusively Homosexual | *χ²* |  |  |  |  |  |  |
|  | **Men** | | |  |  |  |  |  |  |  |  |  |  |  |  |
|  | Current living area (*N* = 1116) | | |  |  |  |  |  |  |  |  |  |  |  |  |
|  |  | Urban | | 42.1% (217) | 47.0% (94) | 45.5% (40) | 42.1% (32) | 58.2% (138)🡩 | 33.52* |  |  |  |  |  |  |
|  |  | Suburban | | 46.6% (240)🡩 | 37.0% (74) | 38.6% (34) | 47.4% (36) | 25.7% (61)🡫 |  |  |  |  |  |  |  |
|  |  | Rural | | 11.3% (58) | 16.0%(32) | 15.9%(14) | 10.5%(8) | 16.0% (38) |  |  |  |  |  |  |  |
|  | Childhood living area (*N* = 1125) | | |  |  |  |  |  |  |  |  |  |  |  |  |
|  | Urban | | | 24.0% (125) | 25.6% (52) | 18.2% (16) | 18.2% (14) | 27.0% (64) | 12.24 |  |  |  |  |  |  |
|  | Suburban | | | 55.6% (289) | 47.3% (96) | 52.3% (46) | 51.9% (40) | 48.1% (114) |  |  |  |  |  |  |  |
|  | Rural | | | 20.4% (106) | 27.1% (55) | 29.5% (26) | 29.9% (23) | 24.9% (59) |  |  |  |  |  |  |  |
|  | Feminist Views (*N* = 1123) | | |  |  |  |  |  |  |  |  |  |  |  |  |
|  | Yes | | | 42.9% (222) | 53.7% (108) | 49.4% (43) | 52.6% (41) | 54.8% (131) | 25.32* |  |  |  |  |  |  |
|  | To Some Extent | | | 40.9% (212) | 38.3% (77) | 41.4% (36) | 43.6% (34) | 36.0% (86) |  |  |  |  |  |  |  |
|  | No | | | 16.2% (84)🡩 | 8.0% (16) | 9.2% (8) | 3.8% (3)🡫 | 9.2% (22) |  |  |  |  |  |  |  |
|  | **Women** | | |  |  |  |  |  |  |  |  |  |  |  |  |
|  | Current living area (*N* =3592) | | |  |  |  |  |  |  |  |  |  |  |  |  |
|  | Urban | | | 37.1% (450)🡫 | 48.2% (492) | 49.1% (401) | 49.8% (108) | 52.3% (170) | 53.60* |  |  |  |  |  |  |
|  | Suburban | | | 46.4% (565)🡩 | 40.1% (409) | 38.9% (318) | 38.7% (84) | 33.5% (109)🡫 |  |  |  |  |  |  |  |
|  | Rural | | | 16.3% (198)🡩 | 11.7% (119) | 12.0% (98) | 11.5% (25) | 14.2% (46) |  |  |  |  |  |  |  |
|  | Childhood living area (*N* = 3606) | | |  |  |  |  |  |  |  |  |  |  |  |  |
|  | Urban | | | 23.3% (285) | 21.2% (216) | 22.9% (187) | 22.2% (48) | 21.8% (72) | 3.32 |  |  |  |  |  |  |
|  | Suburban | | | 51.8% (633) | 54.6% (556) | 52.1% (426) | 55.6% (120) | 52.4% (173) |  |  |  |  |  |  |  |
|  | Rural | | | 24.9% (305) | 24.2% (247) | 25.1% (205) | 22.2% (48) | 25.8% (85) |  |  |  |  |  |  |  |
|  | Feminist Views (*N* = 3613) | | |  |  |  |  |  |  |  |  |  |  |  |  |
|  | Yes | | | 68.7% (838)🡫 | 78.5% (801) | 81.0% (667) | 79.4% (173) | 82.8% (275) | 72.78* |  |  |  |  |  |  |
|  | To Some Extent | | | 26.0% (317)🡩 | 19.4% (198) | 16.4% (135)🡫 | 19.7% (43) | 15.7% (52)🡫 |  |  |  |  |  |  |  |
|  | No | | | 5.3% (65)🡩 | 2.1% (21)🡫 | 2.6% (21) | 0.9% (2) | 1.5% (5) |  |  |  |  |  |  |  |

*Note.* 🡩observed frequency is higher than expected frequency; 🡫 observed frequency is less than expected frequency; **p* < .01

| Table 2S. Results of ANOVA and Tukey Post-Hoc Analysis of Religiosity, Age, and Education Levels | | | | |
| --- | --- | --- | --- | --- |
|  | *df* | *F* | *p* | Tukey |
| **Religiosity Level** |  |  |  |  |
| Men: Median = 1, IR = 1  Women: Median = 1, IR = 1 |  |  |  |  |
| Gender | 1 | 1.29 | .260 | Men: MostHm > ExcHm (*d* = 0.47)*  Women: ExcHt > MstHt (*d* = 0.21)*  ExcHt > Bi (*d* = 0.2)* |
| Sexual Orientation | 4 | 3.39 | .006 |  |
| Gender X Sexual Orientation | 4 | 5.07 | < .001 |  |
| **Education Level** |  |  |  |  |
| Men: Median = 5, IR = 2  Women: Median = 5, IR = 2 |  |  |  |  |
| Gender | 1 | 16.16 | < .001 | Men: ExcHm > MostHm (*d* = 0.43)*  Women: Bi < ExcHt (*d* = 0.23)*  Bi < MstHt (*d* = 0.18)*  Bi < ExcHm (*d* = 0.4)* |
| Sexual Orientation | 4 | 10.42 | < .001 |  |
| Gender X Sexual Orientation | 4 | 1.57 | .180 |  |
| **Age** |  |  |  |  |
| Men: Mean = 35.104, SD = 14.69, Range = 16 - 89  Women: Mean = 33.05, SD = 13.43, Range = 16 - 82 | | | | |
| Gender | 1 | 0.30 | .59 | Men: ExcHt > MstHt (*d* = 0.39) *  ExcHt > MstHm (*d* = 0.52) *  MstHt< ExcHm (*d* = 0.40) *  MstHm < ExcHm (*d* = 0.6) *  Women: ExcHt > MstHT (*d* = 0.53) *  ExcHt > Bi (*d* = 0.79) *  MstHt < ExcHm (*d* = 0.69) *  Bi < MstHm (*d* = 0.36) *  Bi < ExchHm (*d* = 0.92) *  MstHm < ExcHm (*d* = 0.51) * |
| Sexual Orientation | 4 | 47.81 | < .001 |  |
| Gender X Sexual Orientation | 4 | 4.49 | = .001 |  |
|  |  |  |  |  |
|  |  |  |  |  |
|  | | | |  |
|  | | | |  |

IR: Interquartile range; SD: Standard deviation

ExcHt: Exclusively heterosexual; MstHt: Mostly heterosexual; Bi: Bisexual; MstHm: Mostly homosexual; ExcHm: Exclusively homosexual.

* *p* < .01

| Table 3S. Logistic Regression Analysis for predicting binary/non-binary response patterns with gender, sexual orientation and the interaction between them. Exclusively heterosexual men constitute a reference category. | | | | | |
| --- | --- | --- | --- | --- | --- |
|  | B | S.E. | Wald | df | Sig. |
| **Gender** | .16 | .11 | 2.02 | 1 | .16 |
| **Sexual orientation** |  |  | 67.71 | 4 | .00 |
| MstHet | -1.81 | .27 | 47.00 | 1 | .00 |
| Bi | -1.53 | .35 | 19.06 | 1 | .00 |
| MstHom | -1.12 | .33 | 11.54 | 1 | .00 |
| ExcHom | -.22 | .18 | 1.57 | 1 | .21 |
| **Gender X Sexual orientation** |  |  | 14.30 | 4 | .01 |
| Woman X MstHet | .82 | .28 | 8.34 | 1 | .00 |
| Woman X Bi | -.00 | .37 | .00 | 1 | .99 |
| Woman X MstHom | -.21 | .38 | .30 | 1 | .58 |
| Woman X ExcHom | -.39 | .22 | 3.07 | 1 | .08 |
